# Levels of Sex Steroids in Plethodontid Salamanders: A Comparative Study Within the Genus *Aneides*

**DOI:** 10.1002/ece3.70550

**Published:** 2024-11-24

**Authors:** Nancy L. Staub, Stephen G. Hayes, Mary T. Mendonca

**Affiliations:** ^1^ Biology Department Gonzaga University Spokane Washington USA; ^2^ Department of Biological Sciences Auburn University Auburn Alabama USA

**Keywords:** androgen, estrogen, secondary sexual characteristics, sexual dimorphism, sexual monomorphism

## Abstract

Derived monomorphism is a condition in which males and females are phenotypically similar, but the similarity is derived. Derived monomorphism typically evolves from sexual dimorphism or from a different monomorphic state. We examined the hormonal basis of derived monomorphism in the salamander genus *Aneides* (Plethodontidae). We reject our hypothesis that circulating levels of androgens explain the derived traits, such as enlarged jaw musculature, in females (some would call them “male‐like traits”). There was no clear pattern of differences in androgen levels or degree of dimorphism in androgen levels, between the sexually dimorphic *Aneides hardii* and the other, derived monomorphic, species studied. Concentrations of testosterone and dihydrotestosterone were higher in males than in females in all species examined. The degree of sexual dimorphism in androgen level was also consistent among the species studied. Levels of androgens in female plethodontids have not been previously reported.

## Graphical abstract

Species in the salamander genus *Aneides* (family Plethodontidae) show interesting patterns of sexual dimorphism. Levels of androgens in males and females do not explain the variation observed between species in the degree of dimorphism and monomorphism.

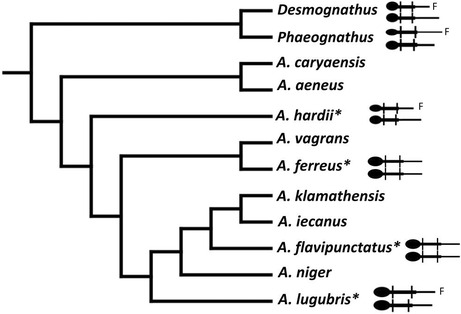

## 1 Introduction

In some species, sexual dimorphism is common and has inspired numerous studies investigating its proximate and ultimate causes (e.g., Cox et al. 2005). In other species, sexual monomorphism is present as a derived state, evolved from a sexual monomorphic or dimorphic condition. This phenomenon, derived monomorphism, has intrigued biologists (e.g., Burns 1998; Cox, Zilberman, and John‐Alder 2008; Galán 2000; Jiménez, Burgos, and Barrionuevo 2023; Staub 2021), but hasn't received as much attention as sexual dimorphism or as sex‐role reversal, a type of dimorphism (Fritzsche et al. 2021). Charles Darwin commented on species in which females display traits that are typically male secondary sexual characteristics and described it as the “transference to the female of the characteristics acquired by the males” (Darwin 1871, 793). Darwin was primarily intrigued with what's known as sex‐role reversal, a pattern in which the ancestral pattern of sexual dimorphism is switched between males and females. Derived monomorphism is related to sex‐role reversal, but in this case both sexes are phenotypically similar to each other, rather than the dimorphic pattern being reversed between the sexes. Derived monomorphism is understudied, perhaps because a phylogenetic context is necessary in order to distinguish the derived pattern from ancestral monomorphism. A recent study describing the morphological patterns of derived monomorphism (or reduced dimorphism) in the salamander genus *Aneides* (Staub 2021) hypothesized that elevated levels of androgens in females are the proximate mechanism for the expression of these derived traits in females and thus of the reduced dimorphism. Our study tests this hypothesis by examining levels of androgens in several species of plethodontid salamanders.

Our hypothesis is based on examples in which female morphology or behavior are due to androgenic action. For example, levels of testosterone were higher in females of the polyandrous black coucal (*Centropus grillii*) than in females of socially monogamous species (Goymann 2023). In the polyandrous species, females are larger than males and defend territories, while males incubate eggs (Goymann 2023). As another example, female Edible frogs (*Pelophylax esculentus*) have relatively high levels of androgens, higher even than males, during part of the seasonal cycle and possess skin glands that are testosterone dependent (Delrio et al. 1979; d'Istria et al. 1982). In mammals, female Iberian moles develop androgenized external genitalia, increased muscle mass, and display aggressive behavior due to ovotestes that produce relatively high levels of androgens (Real et al. 2020). Another mammalian example is the spotted hyena *Crocuta crocuta*, in which females are hypothesized to be androgenized at an early age (reviewed by Place and Glickman 2004; Jiménez, Burgos, and Barrionuevo 2023). In addition to testosterone being important to female development as a precursor to estradiol, these examples indicate androgens play important roles in normal female development as well (see Staub and DeBeer 1997, for a review).

In other cases, however, levels of androgens are not sufficient to explain derived secondary sexual traits in females, in either derived monomorphic or sex‐role reversed species. Lipshutz and Rosvall (2020a) reject the hypothesis that activational effects of androgens explain sex‐role reversal and hypothesize instead that organizational effects and sex‐specific differences in androgen sensitivities are more promising explanations. For example, in many sex role‐reversed birds such as the Northern Jacana (*Jacana spinosa*), levels of testosterone do not fully explain differences between the sexes (Lipshutz and Rosvall 2020b).

**FIGURE 1:**
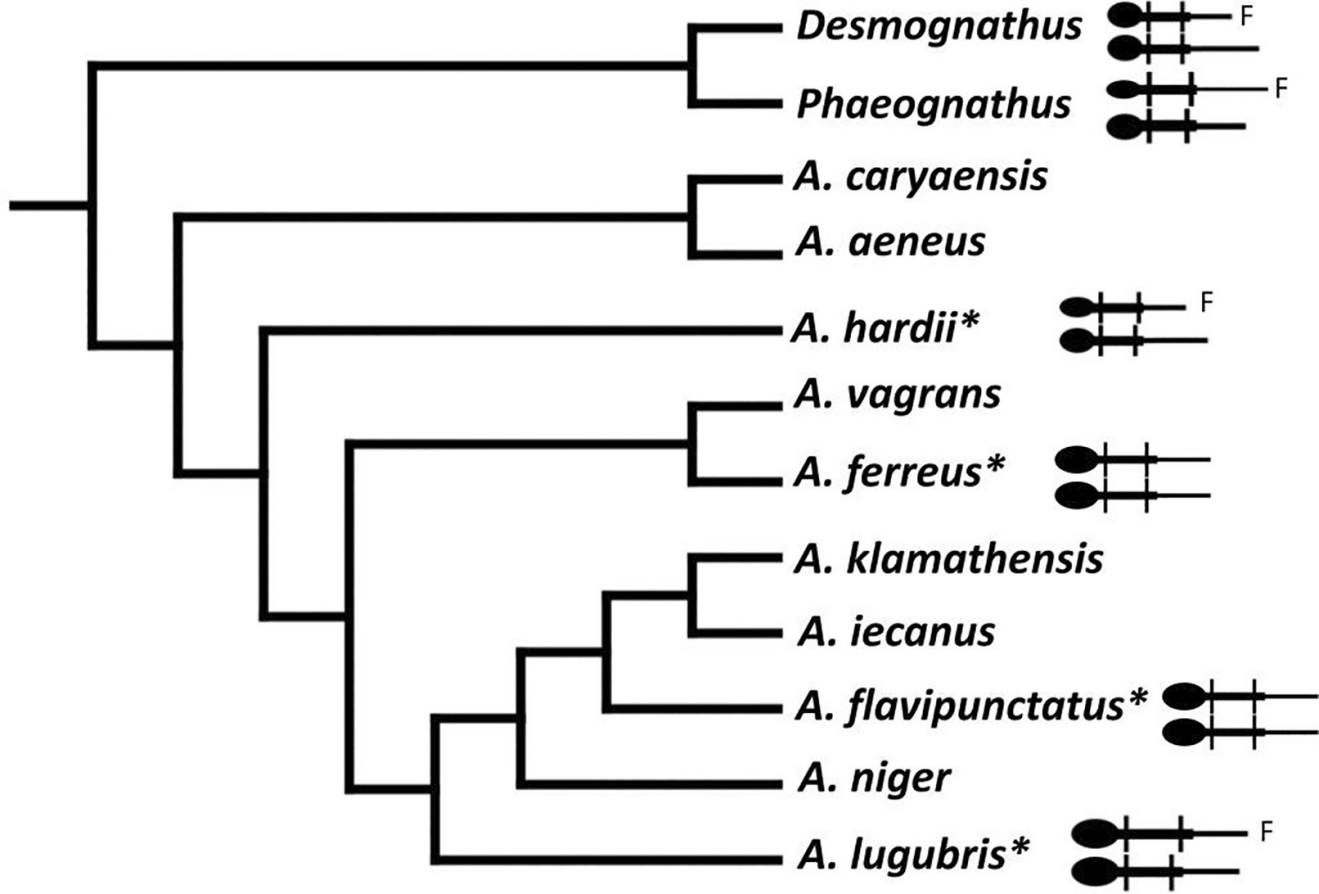
Phylogeny of the genus *Aneides*, adapted from Staub (2021) and based on Vieites, Min, and Wake (2007), Shen et al. (2016), Patton et al. (2019), Reilly and Wake (2019), and Jackman (1998). When dimorphism in length is present, an “F” indicates the female. Head width dimorphism is indicated by different sized heads. An * indicates those species included in our study.

Members of the genus *Aneides* (Plethodontidae) are direct developers and terrestrially active in rainy or humid seasons. Fertilized ova are typically deposited in grape‐like clusters in a damp space, such as within a decaying log, underground, or in a tree cavity. Females stay with the embryos throughout development, and they can remain in loose family groups post‐hatching as well, with males reported in attendance occasionally (Stebbins 1951). This genus is characterized by dramatic morphological variation both within and between species and by a number of derived morphological traits (Wake 1966; Larson et al. 1981). In addition to expressing morphological novelty relative to its outgroups, the species of *Aneides* show varying levels of sexual dimorphism (Figure 1; Larson et al. 1981; Staub 2021). *Aneides hardii*, an isolated species in the Sacramento and Lincoln Mountains of New Mexico, is sexually dimorphic in body size and head width, with males being larger and having wider heads than females. Females and males of other species of the genus express traits that are typical of male *A. hardii* (e.g., hypertrophied jaw muscles, large snout‐vent length) and thus are less sexually dimorphic than *A. hardii* (Staub 2021). The outgroup *Desmognathus* shows dimorphism in body size in most species, with males larger than females (Bruce 1993; Camp, Soelter, and Wooten 2019). The other outgroup, *Phaegnathus hubrichti*, has male‐biased dimorphism as well (Bakkegard and Guyer 2004). This comparison indicates that the male‐biased size dimorphism observed in *A. hardii* is ancestral for *Aneides*; the absence of size dimorphism in some species of *Aneides* then is derived monomorphism. The ancestral condition for head width dimorphism is not as clear. While *P. hubrichti* and some species of *Desmognathus* show dimorphism in head width (Bakkegard and Guyer 2004; Bakkegard and Rhea 2012), more recent work did not detect head dimorphism in species of *Desmognathus* (Camp, Soelter, and Wooten 2019). Furthermore, the specific features involved in enlarged heads of *Desmognathus* differ from those in *Aneides* (Schwenk and Wake 1993). More work is needed here to resolve these character states for head dimorphism.

The traits involved in head width dimorphism in *Aneides*, for example hypertrophied jaw muscles, are secondary sexual characteristics, typically beginning development at sexual maturity (Staub 2016). Secondary sexual characteristics are regulated by androgens (Norris and Carr 2021) and the relatively few secondary sexual traits that have been studied in salamanders are androgen dependent (Noble and Pope 1929; Schubert et al. 2006; Sever 1976; Stewart 1958; Woodley 1994; Zimmer and Dent 1981). We aim to examine androgen levels in this morphological and phylogenetic context. The phylogeny of *Aneides* has been well studied (Figure 1) but some relationships are unclear as speciation is thought to have occurred relatively quickly (Mahoney 2001; Reilly and Wake 2019; Vieites, Min, and Wake 2007).

We hypothesize that females in species which express derived monomorphism (*Aneides lugubris*, *Aneides ferreus*, *Aneides flavipunctatus*) have higher levels of androgens compared to females of other species that lack these traits (*A. hardii*). In addition to increasing our understanding of the role of androgens in females, our results are important because little is known about androgen levels in plethodontid salamanders. Because of this lack of information, we also include data for *Plethodon elongatus*, a species in the western clade of *Plethodon*.

## 2 Methods

### 2.1 Sample Collection

Individuals of *A. lugubris*, *A. ferreus*, *A. flavipunctatus*, *A. hardii*, and *P. elongatus* were hand collected in the field (see Table 1 for sample sizes and Appendix 1 for localities and dates), sacrificed by decapitation within 3 min of capture, and blood samples collected in heparinized capillary tubes and stored on ice. Within 3 h of collection, blood samples were centrifuged and the plasma frozen. Samples were kept at −80°C until radioimmunoassays (RIAs) were performed. Animals were fixed in 10% neutral buffered formalin, rinsed in water, and stored in 70% ethanol. Sex was determined via gonad inspection. One testis was removed from males for histological analysis. Authorization for this work included: Oregon Dept of Fish and Wildlife permits 437‐93, 051‐94, 038‐95, 045‐96; New Mexico Dept of Game and Fish permit 1702 (1993–1996). California Department of Fish and Game permits 4023 1993–1995, 4755 1995–1997.

**TABLE 1:** Sample sizes for mature (a), and immature (b) animals by species and month.

Species	Month	Female	Male
(a)			
*A. hardii*	May	7	6
*A. hardii*	June	3	10
*A. hardii*	July	5	14
*A. hardii*	August	8	2
*A. lugubris*	January	4	3
*A. lugubris*	February	2	6
*A. lugubris*	March	2	6
*A. lugubris*	November	1	4
*A. ferreus*	January	4	3
*A. ferreus*	February	1	5
*A. ferreus*	March	2	5
*A. ferreus*	May	3	7
*A. ferreus*	August	2	4
*A. ferreus*	October	0	2
*A. ferreus*	November	2	4
*A. ferreus*	December	7	15
*A. flavipunctatus*	March	4	8
*P. elongatus*	January	1	3
*P. elongatus*	March	0	3
*P. elongatus*	November	1	0
(b)			
*A. hardii*	May	4	2
*A. hardii*	June	3	2
*A. hardii*	July	3	0
*A. ferreus*	January	0	2
*A. ferreus*	February	2	1
*A. ferreus*	March	5	3
*A. ferreus*	May	0	2
*A. ferreus*	August	2	2
*A. ferreus*	October	2	0
*A. ferreus*	November	4	1
*A. ferreus*	December	6	2
*A. lugubris*	February	1	1
*A. lugubris*	March	2	0
*A. lugubris*	November	1	0

Blood samples were collected across the animal's active season: *A. lugubris* (October–May), *A. ferreus* (September—May), and *A. hardii* (May–August). *A. flavipunctatus* samples were collected in March only. For *P. elongatus*, samples were collected over several months (October, November, January, March), but sample sizes were too low to analyze seasonally.

### 2.2 Radioimmunoassays

Plasma Estradiol (E), testosterone (T), and dihydro‐testosterone (DHT), were separated using column chromatography and measured by radioimmunoassay (RIA). Extraction, Celite microcolumn chromatography, and radioimmunoassay techniques followed the protocol in Wingfield, Smith, and Farner (1982). Basically, plasma samples were equilibrated overnight with 800–1000 cpm of each steroid hormone to be measured to determine recovery efficiency. Plasma was extracted with diethyl ether, dried under nitrogen gas, and resuspended in 10% ethyl acetate in iso‐octane. Samples were chromatographed on microcolumns of Celite:propylene/ethylene glycol. The eluate solvents used were 10% ethyl acetate in iso‐octane for DHT, 20% ethyl acetate in iso‐octane for T, and 40% ethyl acetate in iso‐octane for E. Column aliquots were dried under nitrogen and resuspended in phosphate buffer. Two aliquots were taken (1, 1:20 for DHT, and T, and duplicates for E). Aliquots were incubated overnight at 4°C with the tritiated steroid to be measured and its respective antibody (E antibody, 17‐94 from Endocrine Sciences, Tarzana, CA; androgen antibody, T3003). Following the incubation period, the assay tubes received a 5% charcoal: 0.5% dextran mixture to remove unbound steroid. Tubes were vortexed, incubated for 15 min at 4°C, and centrifuged for 10 min at 3000 rpm. The supernatant was collected from each sample, equilibrated overnight, and counted in the Beckman scintillation counter the following day (see Navara et al. 2006; Wingfield, Smith, and Farner 1982).

Sensitivities were 15 pg/mL for T, DHT, and E. Inter‐assay coefficients of variation were E: 13% (*n* = 11), T: 10% (*n* = 11), DHT: 8% (*n* = 10). Values for intra‐assay variation were E: 8% (*n* = 138), T: 7% (*n* = 150), DHT: 8% (*n* = 131).

### 2.3 Histology

Using standard histological procedures, testes were embedded in paraffin, sectioned at 12 μm, mounted on slides, and stained with hematoxylin and eosin (Presnell and Schreibman 1997).

### 2.4 Statistical Analysis

To examine our hypothesis, we modeled sex steroid concentration as a function of sex and species for *A. hardii*, *A. ferreus*, *A. flavipunctatus*, *A. lugubris*, and *P. elongatus* (i.e., five‐species model). Because little has been published on sex steroid variation in these species, we first modeled E, T, and DHT concentration as a function of month and sex, to determine which months to include in the five‐species model. We first fit interaction models of month by sex for adult *A. hardii*, *A. ferreus*, and *A. lugubris*. If interactive effects were not significant, we dropped the interaction term, refit, and interpreted the main effects models of month and sex. Our *P. elongatus* and *A. flavipunctatus* sample sizes were too small to fit sex by month models.

Following our interpretation of the sex by month models (see Results, Seasonal Variation), our five‐species model included June, July, and August observations for *A. hardii*, January, February, March, and November observations for *A. lugubris*, and January, February, March, May, August, October, November, and December observations for *A. ferreus*, March observations for *A. flavipunctatus*, and January, March, and November observations for *P. elongatus*. Our hypothesis predicted we would observe higher levels of T and DHT in males than in females of the sexually dimorphic *A. hardii*, but higher levels of T and DHT in female *A. ferreus*, *A. flavipunctatus*, and *A. lugubris*, compared to female *A. hardii*, due to derived monomorphism. We therefore expected to observe a large sex by species interaction in the five‐species model, with pairwise comparisons of sex within species revealing different levels of T and DHT in *A. hardii*, and less of a difference in *A. ferreus*, *A. flavipunctatus*, and *A. lugubris*.

Because little is known about sex steroid variation in these species, we also report results of modeling E, T, and DHT as a function of sex and species in immature *A. hardii* and *A. ferreus*. Sample sizes for immature *A. lugubris* were too low to be included in the modeling analyses.

For all models, we included data with and without potential outliers, as suggested by Pollet and van der Meij (2017). We identified potential outlier observations as those more than 1.5 interquartile range units away from the median, within sex by month groups. Modeling results were equivalent with and without outliers, so we retained all observations for modeling. We log‐transformed our hormone observations to stabilize variance and analyzed residuals to evaluate model fit. We made post hoc comparisons using the Tukey HSD test. We also provide summary statistics for individual T/DHT ratios by sex and species, and mean male/female T and DHT ratios by species. All analysis was done using R (R Core Team 2023).

## 3 Results

### 3.1 Seasonal Variation

For adult male *A. ferreus*, sperm were found in the vas deferens in every month examined. This corroborates results by McKenzie and Storm (1970) that found sperm in all months except September. Male *A. lugubris* shows a pattern similar to that of *A. ferreus*: sperm were found in the vasa throughout the active season. In contrast to *A. ferreus* and *A. lugubris*, no sperm were found in the vasa of *A. hardii* males during their active season, June through September. This pattern of no sperm in the vasa at this time was documented by Williams (1978) as well.

**FIGURE 2:**
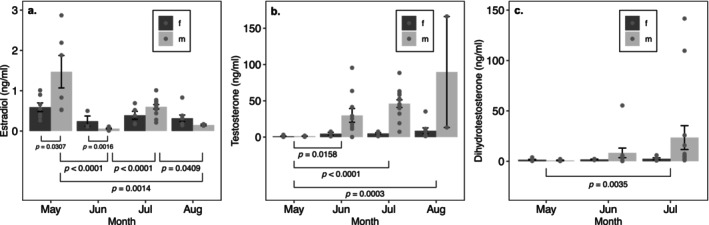
Effects of sex and month on hormone levels in mature *A. hardii*. Points indicate individual observations, and bar height indicates mean (±SE) hormone level. Sex and month had an interactive effect on estradiol (a), while month had only main effects on testosterone (b), and dihydrotestosterone (c). Brackets and *p*‐values illustrate results of post hoc Tukey comparisons.

Seasonal variation in E, T, and DHT was apparent for *A. hardii* (Figure 2). We observed interactive effects of month and sex on E levels in *A. hardii* (*p* = 0.0008) (Table 2a). In males, E levels were greater in May than June or August (*p* < 0.0001 and *p* = 0.0014, respectively), and greater in July than June or August (*p* < 0.0001 and *p* = 0.0409, respectively) (Figure 2a). We did not observe large variation in E levels in females over the months examined (*p* > 0.05). We also did not observe interactive effects of sex and month on T levels in *A. hardii* (*p* = 0.2177), but we did observe significant month and sex main effects (*p* < 0.0001 and *p* = 0.0010, respectively) (Table 2b). In females and males, T levels were lower in May than in June (*p* = 0.0158), July (*p* < 0.0001), or August (*p* = 0.0003) (Figure 2b). Since May was different from the other months, we omitted May *A. hardii* observations from the five‐species model. In addition, T levels were consistently greater in males than females (*p* = 0.0010). Interactive effects of month and sex on DHT were not significant (*p* = 0.1383), nor were sex main effects (*p* = 0.3077) (Table 2c). Month main effects were significant, however (*p* = 0.0052). In females and males, DHT levels were lower in May than in July (*p* = 0.0035).

**TABLE 2:** ANOVA tables for 2‐way models of sex and month effects on adult *A. hardii* estradiol (a), testosterone (b), and dihydrotestosterone (c).

	SS	df	*F*	*p*
(a) Estradiol				
Sex	2.036	1	4.96	0.031
Month	3.001	3	2.43	0.076
Sex * Month	8.618	3	6.58	0.0008
Residuals	19.725	48		
(b) Testosterone				
Sex	21.813	1	12.28	0.0010
Month	65.875	3	12.37	< 0.0001
Residuals	88.782	50		
(c) Dihydrotestosterone				
Sex	1.857	1	1.02	0.32
Month	21.372	2	5.85	0.0058
Residuals	74.916	41		

*Note:* Initial models contained interactions, which were dropped from final models when not significant (*p* > 0.05).

**FIGURE 3:**
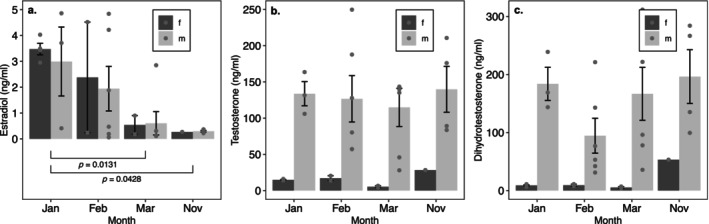
Effects of sex and month on hormone levels in mature *A. lugubris*. Points indicate individual observations, and bar height indicates mean (±SE) hormone level. Sex and month had main effects on estradiol (a), while sex had only main effects on testosterone (b), and dihydrotestosterone (c). Brackets and *p*‐values illustrate results of post hoc Tukey comparisons.

Seasonal variation in E, T, and DHT was also apparent for *A. lugubris*, but we did not observe a clear pattern of month effects (Figure 3). We did not observe interactive effects of sex and month on E (*p* = 0.9820), but main effects of month were significant (*p* = 0.0119) (Table 3a). In males and females, E levels were greater in January than March (*p* = 0.0131) or November (*p* = 0.0428) (Figure 3a). We also did not observe interactive effects of sex and month on T (*p* = 0.2584) or DHT (*p* = 0.0957), nor were month main effects significant on T (*p* = 0.1989) or DHT (*p* = 0.0829) (Table 3b,c). Sex did have significant effects on T and DHT, however (Figure 3b,c). Across all months, males had greater T (*p* < 0.0001) and DHT (*p* < 0.0001) levels than females.

**TABLE 3:** ANOVA tables for 2‐way models of sex and month effects on adult *A. lugubris* estradiol (a), testosterone (b), and dihydrotestosterone (c).

	SS	df	*F*	*p*
(a) Estradiol				
Sex	0.884	1	0.60	0.45
Month	20.214	3	4.57	0.011
Residuals	33.940	23		
(b) Testosterone				
Sex	26.533	1	85.10	< 0.0001
Month	1.572	3	1.68	0.20
Residuals	7.7171	23		
(c) Dihydrotestosterone				
Sex	36.232	1	79.08	< 0.0001
Month	3.467	3	2.52	0.083
Residuals	10.538	23		

*Note:* Initial models contained interactions, which were dropped from final models when not significant (*p* > 0.05).

**FIGURE 4:**
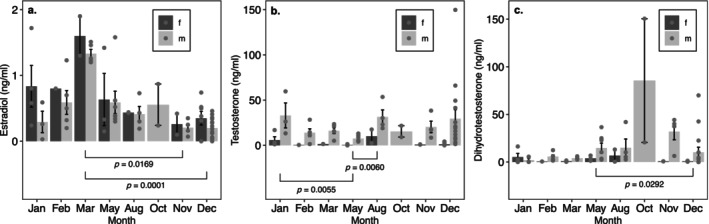
Effects of sex and month on hormone levels in mature *A. ferreus*. Points indicate individual observations, and bar height indicates mean (±SE) hormone level. Only month had main effects on estradiol (a), while sex and month had main effects on testosterone (b), and dihydrotestosterone (c). Brackets and *p*‐values illustrate results of post hoc Tukey comparisons.

Seasonal variation in E, T, and DHT was also apparent for *A. ferreus*, but there was no clear pattern of month effects (Figure 4). We did not observe interactive effects of sex and month on E (*p* = 0.8925), nor were sex main effects significant (*p* = 0.3590), but main effects of month were significant (*p* = 0.0002) (Table 4a). In males and females, E levels were greater in March than November (*p* = 0.0169) or December (*p* = 0.0001) (Figure 4a). We also did not observe interactive effects of sex and month on T (*p* = 0.4017) or DHT (*p* = 0.4203). Month did have significant main effects on T (*p* = 0.0046) and DHT (*p* = 0.0043), however (Table 4b,c). For both sexes, levels of T were greater in January than May (*p* = 0.0055) and lower in May than in August (*p* = 0.0060) (Figure 4b). Levels of DHT for both sexes were greater in May than December (*p* = 0.0292) (Figure 4b,c). Across all months, males had greater levels of T (*p* < 0.0001), but DHT was not different between *A. ferreus* males and females (*p* = 0.3389).

**TABLE 4:** ANOVA tables for 2‐way models of sex and month effects on adult *A. ferreus* estradiol (a), testosterone (b), and dihydrotestosterone (c).

	SS	df	*F*	*p*
(a) Estradiol				
Sex	0.852	1	0.86	0.36
Month	34.437	7	4.94	0.0002
Residuals	56.789	57		
(b) Testosterone				
Sex	116.050	1	91.52	< 0.0001
Month	29.792	7	3.36	0.0046
Residuals	72.281	57		
(c) Dihydrotestosterone				
Sex	4.468	1	0.93	0.34
Month	114.015	7	3.39	0.0043
Residuals	273.686	57		

*Note:* Initial models contained interactions, which were dropped from final models when not significant (*p* > 0.05).

### 3.2 Sexual Dimorphism

**FIGURE 5:**
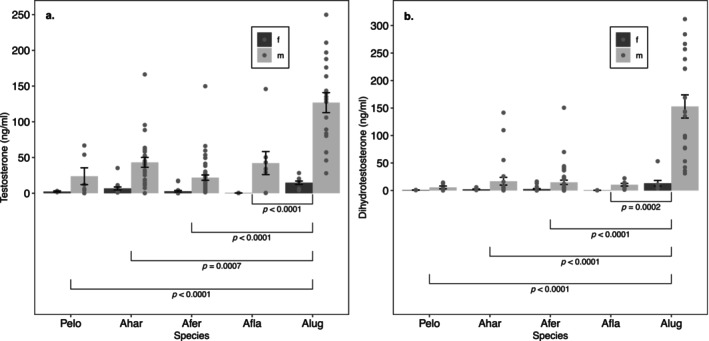
Effects of sex and species on hormone levels in mature *P. elongatus*, *A. hardii*, *A. ferreus*, *A. flavipunctatus*, and *A. lugubris*. Points indicate individual observations, and bar height indicates mean (±SE) hormone level. Sex and species had main effects on testosterone (a), and dihydrotestosterone (b). All species are sexually dimorphic for T and DHT concentrations, and the degree of dimorphism is consistent between species (no interaction effect). Within *Aneides*, *A. hardii* is the species with the greatest morphological sexual dimorphism, and the others show less dimorphism. Brackets and *p*‐values illustrate results of post hoc Tukey comparisons.

We did observe significant main effects for sex and species‐‐concentrations of T and DHT are sexually dimorphic (*p* < 0.0001) (Table 5) and the degree of dimorphism was consistent across species; there was no significant sex by species interactions in models of either T (*p* = 0.0862) or DHT (*p* = 0.2165) (Figure 5). There was sexual dimorphism in androgen levels across species; males had greater levels of both T (mean difference in ln (T) = 2.34 ng/mL, SE = 0.231) and DHT (mean difference in ln (T) = 1.42 ng/mL, SE = 0.344) than females. Androgen levels were also significantly different among the five species (*p* < 0.0001). *A. lugubris* (male and female) had greater T and DHT levels than *P. elongatus* (*p* = 0.0002 and *p* < 0.0001, respectively), *A. hardii*, *A. ferreus*, and *A. flavipunctatus* (all with *p* < 0.0001) (Figure 5a,b). Our prediction that *A. hardii* males would have greater androgen levels than females is supported. However, our prediction that female *A. lugubris*, *A. ferreus*, and *A. flavipunctatus* would have higher androgen levels than female *A. hardii* is not supported.

**TABLE 5:** ANOVA tables for 2‐way models of sex and species effects on adult *P. elongatus, A. hardii, A. ferreus, A. flavipunctatus and A. lugubris testosterone* (a), and dihydrotestosterone (b).

	SS	df	*F*	*p*
(a) Testosterone				
Sex	189.39	1	102.51	< 0.0001
Species	120.12	4	16.25	< 0.0001
Residuals	277.11	150		
(b) Dihydrotestosterone				
Sex	61.47	1	16.94	< 0.0001
Species	241.78	4	16.65	< 0.0001
Residuals	511.80	141		

*Note:* Initial models contained interactions, which were dropped from final models when not significant (*p* > 0.05).

A careful reader may note that the five‐species model discussed here presents different results compared to the analysis by species in the seasonal results above. For example, in the 5‐species model, we conclude that levels of DHT are sexually dimorphic when DHT is different when averaged over the levels of all five species, whereas for *A. ferreus* in the seasonal test, no difference was observed between males and females. In the 5‐species model, there is enough sexual dimorphism of DHT in the other four species that the effect of the overlapping variation (between the sexes) in *A. ferreus* is swamped by the non‐overlapping variation in the other species. Another way to explain this is by looking at the mean difference in DHT between males and females for *A. ferreus* alone (0.575, SE = 0.596, on the log scale) and looking at the mean difference in DHT between males and females for all five species (1.32, SE = 0.32 on the log scale). The 5‐species mean difference in DHT between the sexes is larger, and the hypothesis test is more powerful (i.e., the SE is smaller). From a statistical point of view, this difference in reporting patterns of sexual dimorphism makes sense.

### 3.3 Levels of Androgens in Immatures

**FIGURE 6:**
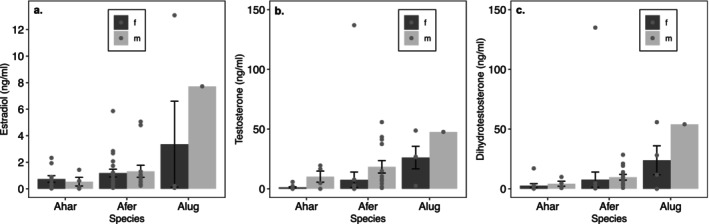
Effects of sex and species on hormone levels in immature *A. hardii*, *A. ferreus*, and *A. lugubris*. Points indicate individual observations, and bar height indicates mean (±SE) hormone level. *Aneides lugubris* was omitted from statistical analysis due to small sample sizes. Estradiol levels were greater for *A. ferreus* than *A. hardii* (*p* = 0.0223), but species effects were not significant for testosterone or dihydrotestosterone. Sex effects were, however, significant for testosterone (*p* = 0.0001) and dihydrotestosterone (*p* = 0.0003).

Small sample sizes for immature individuals only allowed us to fit interactive models of sex and species for *A. hardii* and *A. ferreus* (Figure 6; Table 6). Interactions were not significant for E (*p* = 0.3307), T (*p* = 0.3445), or DHT (*p* = 0.6749). E levels in immature animals, however, were greater in *A. ferreus* than in *A. hardii* (*p* = 0.0223), regardless of sex. Species effects were not large for T (*p* = 0.3170) or DHT (*p* = 0.1716), but in both species levels of these androgens were greater for males than females (T: *p* = 0.0001, DHT: *p* = 0.0003).

**TABLE 6:** ANOVA tables for 2‐way models of sex and species effects on immature *A. hardii*, and *A. ferreus* for estradiol (a), testosterone (b), and dihydrotestosterone (c). Initial models contained interactions, which were dropped from final models when not significant (*p* > 0.05).

	SS	df	*F*	*p*
(a) Estradiol				
Sex	0.176	1	0.12	0.74
Species	8.558	1	5.61	0.022
Residuals	68.704	45		
(b) Testosterone				
Sex	74.490	1	17.40	0.0001
Species	4.385	1	1.024	0.32
Residuals	192.70	45		
(c) Dihydrotestosterone				
Sex	24.177	1	14.62	< 0.0001
Species	3.192	1	1.93	0.17
Residuals	74.421	45		

## 4 Discussion

Our hypothesis, that circulating levels of androgens are higher in females of species showing derived monomorphism than in females of the dimorphic species *A. hardii*, is not supported. That is, sexual dimorphism in androgen levels was not reduced in derived monomorphic species. Sexual dimorphism in T levels was apparent in all species studied, with males having higher levels than females; dimorphism in T levels was not less in the derived monomorphic species (*A. ferreus*, *A. flavipunctatus*, *and A. lugubris*) than in *A. hardii*, the most sexually dimorphic species. Similarly, for levels of DHT, females had lower levels than males in all species studied. Comparing females across different species, absolute levels of androgens were not greater in females of derived monomorphic species. Thus, levels of androgens do not correlate with the observed patterns of dimorphism. However, it is interesting to note that male and female *A. lugubris* had higher androgen levels than the other species. This is the start of another interesting chapter of the story of *A. lugubris*, as this species shows the most extreme development of derived secondary sexual traits, for example, co‐ossification of the skull and skin, and hypertrophied jaw musculature (Wake, Wake, and Wake 1983).

For comparative use in future studies on androgen levels in other amphibian species, we report ratios of male: female T and DHT levels as well as T:DHT ratios (Table 7a,b). A meta‐analysis on birds found that T:DHT ratios did not reflect patterns of sexual dimorphism across 51 species (Goymann and Wingfield 2014). More work on hormone ratios in amphibians will be valuable to identify any patterns in this group. In our case, while our hypothesis of elevated androgen levels in females of species with derived monomorphism was not supported (except perhaps for only *A. lugubris*), our results are interesting nonetheless; we contribute to the few studies on plethodontid steroids (Table 8) and report for the first time levels of androgens in female plethodontids.

**TABLE 7:** Ratios of T/DHT (a) and male: Female ratios of T and DHT. (a) For T/DHT ratios, ratios were formed for each individual and then the mean taken for each species. (b) The means were calculated for T and DHT for each sex within each species and then the mean male: Female ratio was calculated.

Species	Sex	T/DHT	SD	*N*	SE
(a)					
Pelo	f	3.58	3.16	2	2.23
Pelo	m	3.64	1.34	6	0.55
Ahar	f	2.41	2.61	15	0.67
Ahar	m	7.82	7.40	30	1.35
Afer	f	1.61	2.11	20	0.47
Afer	m	59.20	206.78	39	33.11
Afla	f	0.95	0.53	4	0.26
Afla	m	6.02	7.28	8	2.57
Alug	f	1.50	0.60	9	0.20
Alug	m	1.22	0.91	19	0.21

Species	M: FT ratio	M: F DHT ratio
(b)		
Pelo	10.21	6.86
Ahar	10.28	7.61
Afer	9.66	6.21
Afla	125.03	27.84
Alug	8.61	11.62

*Note:* Pelo = *P. elongatus*, Ahar = *A. hardii*, Afer = *A. ferreus*, Afla = *A. flavipunctatus*, and Alug = *A. lugubris*.

**TABLE 8:** Androgen measurements in non‐plethodontids.

Species	Sex	T (ng/mL)	DHT (ng/mL)	Total androgen (ng/mL)	References
*Taricha granulosa*	M			50	Specker and Moore (1980)
*Triturus carnifex*	M			28	Zerani et al. (1991)
*Triturus carnifex*	F			4.5	Zerani et al. (1991)
*Salamandra salamandra*	M	33	2.6	100	Garnier and Joly (1991), Lecouteux et al. (1985)
*Salamandra salamandra*	F	17	2.25		Garnier and Joly (1991)
*Pleurodeles waltl*	M			57.5^a^, 25.3^b^, 43^c^	Cayrol, Garnier, and Deparis (1985); Garnier (1985a)
*Pleurodeles waltl*	F			9.6^a^, 3.5^b^,14^c^	Cayrol, Garnier, and Deparis 1985, Garnier (1985b)
*Ambystoma opacum*	M	50	18		Houck et al. (1996)
*Ambystoma maculatum*	M	16–24			Cooperman, Reed, and Romero (2004)
*Hynobius*	M			320	Hasumi, Iwasawa, and Nagahama (1993)
*Cryptobranchus alleganiensis*	M	100, 95	55, 34.7		Galligan et al. (2021), Case et al. (2024)
*Cryptobranchus alleganiensis*	F	25	12		Galligan et al. (2021)
*Necturus maculosus*	M	70.4	38.7		Bolaffi and Callard (1979)
*Necturus maculosus*	F			7	Bolaffi and Callard (1979)

^a^ Diploid.

^b^ Triploid.

^c^ Ploidy not specified.

Steroid levels in juveniles suggest there may be a critical period in the development in these salamanders where levels of androgens play a key role in development of dimorphic and derived monomorphic traits, but more work is needed to understand these patterns. In *A. flavipunctatus*, head‐width growth rates in males are higher at puberty compared to female growth rates (Staub 2016), although in our study we were not able to examine levels in individuals transitioning from immature to mature *A. flavipunctatus*. Our study is a first step in understanding the complex interplay between hormone levels and the male and female derived monomorphic phenotype.

Of course the relationship between circulating androgen levels and morphology is complex. While the levels of androgens show no clear correlation with the dramatic jaw muscle hypertrophy in *Aneides*, androgen levels are just one factor among many that influence development and contribute to the degree of sexual dimorphism. Alternative hypotheses include that females of derived monomorphic species have higher sensitivities to androgens in target tissues, or different critical enzyme profiles in target tissues (e.g., reductase and aromatase), compared to females that lack these derived secondary sexual characteristics. In addition, T is converted to DHT primarily in the target tissues themselves, so measuring levels in target tissues would be informative here. Another potentially critical variable is the timing of androgen signaling during ontogeny. Investigating these other factors in males and females within and across species in a phylogenetic context will more fully address both the specific question of derived monomorphism in this group of plethodontids as well as contributing to understanding the complex relationship between androgen action and expressed phenotype.

Differential sensitivity to testosterone, rather than level, is hypothesized to be responsible for the derived plumage in female barred buttonquails (Muck and Goymann 2011). The observation that females of sexually dimorphic species respond to androgens (e.g., Blair 1946 on *Bufo fowleri*; Sever 1976 on *Eurycea quadridigitata*; Cox et al. 2015 on *Anolis sagrei*) and develop typical male‐like secondary traits, suggests that slight changes in circulating levels of androgens over evolutionary time could result in major morphological changes without complex changes in gene expression.

Levels of androgens in species of *Aneides* are high compared to most other species of salamanders but do fall within the range of other plethodontid species. For example, in male *Plethodon cinereus*, levels of T reached a maximum of 300 ng/mL (Church and Okazaki 2002). In male *Plethodon shermani*, mean values of T and DHT over time ranged from 98–350 ng/mL and 4–18 ng/mL, respectively (Woodley 1994). And in male *Desmognathus ochrophaeus*, levels of T ranged up to 350 ng/mL and DHT up to 65 ng/mL (Woodley 1994). Note that the reported values above are all for males. Levels of androgens tend to be lower in non‐plethodontids (Table 8).

What's missing from the literature, up until now, are reports of levels of androgens in female plethodontids. Including females in this work on androgen levels and tissue sensitivity is important to understand the role of androgens in development. So while it is tempting to claim that the genus *Aneides* is unusual in that both males and females have relatively high levels of androgens, particularly of DHT, without studies that include females, we just don't know if these levels are unusual.

The role of androgens in female plethodontids has been investigated in a few studies. Early work showed that female *Eurycea quadridigitata* responds to testosterone implants by developing the typical male mental gland and other secondary sexual characteristics (Sever 1976). More recently, Gunelson, Tuong, and Staub (2024) identified androgen receptors in both female and male tail glands in the plethodontid *Desmognathus brimleyorum*, which suggests these tail glands are a secondary sexual characteristic in both sexes. This is similar to nuptial pad development in the frog species *Xenopus laevis* and *Pseudacris triseriata*; both males and females have androgen receptors in the nuptial pad area of the thumb as well as in skin glands in this region (Emerson et al. 1999). More studies like these as well as those examining the activity of androgen metabolic enzymes in target tissues (e.g., Guerriero et al. 2000) are promising areas of research.

As Lipshutz et al. (2019) highlight, the focus on steroid signals themselves, which can be relatively easily measured, over‐simplifies steroid signaling and more specifically oversimplifies the development of secondary sexual traits. What is needed is a more integrated approach that considers all components of the steroid signaling system, including sensitivities, conversions, interaction with other hormones, and the complex inter‐related downstream effects (Lipshutz et al. 2019; Ogino et al. 2011). Promising work in comparing gene expression profiles between males and females from different tissues over time (e.g., Martyniuk, Bissegger, and Langlois 2014; Herrboldt et al. 2021) is an intriguing area of research that may help address the underlying genetic basis, regulation, and development of secondary characteristics in males and females. Studies that examine how steroid signaling may interact with growth factor pathways will help complete the picture as well (e.g., Ogino et al. 2011). Our study is a first step in understanding the complex interplay between hormone levels and the male and female derived phenotype in the genus *Aneides*.

## Author Contributions

**Nancy L. Staub:** conceptualization (lead), data curation (lead), funding acquisition (lead), investigation (equal), methodology (equal), project administration (lead), writing – original draft (equal), writing – review and editing (equal). **Stephen G. Hayes:** formal analysis (equal), methodology (equal), writing – original draft (supporting), writing – review and editing (supporting). **Mary T. Mendonca:** formal analysis (equal), funding acquisition (supporting), investigation (equal), methodology (equal), project administration (supporting), writing – original draft (supporting).

## Conflicts of Interest

The authors declare no conflicts of interest.

## Data Availability

Data and R code used in analyses are available at (DOI): 10.5061/dryad.3xsj3txq5.

## Appendix 1

### 1.1

**Table:**

ID number^a^	Species	Location	Date^b^
1450–1458	*A. ferreus*	S Pistol River on old H101, Curry Co., OR	12‐21‐1993
1459, 1461–1468	*A. ferreus*	S Pistol River on old H101, Curry Co., OR	2‐19‐1994
1491, 1493, 1495–1504	*A. ferreus*	S Pistol River on old H101, Curry Co., OR	5‐12‐1994
1569	*A. ferreus*	Hunter Creek, on FS road 3680. T37S, R13W, E center of section 8. Curry Co., OR	11‐18‐1995
1570–72	*A. ferreus*	FS road 109, T36S, R13W NW corner section 20; Curry Co., OR	11‐17‐1995
1573–1579	*A. ferreus*	S Pistol River on Carpenterville Rd, Pistol River, Curry Co., OR	11‐18‐1995
1585–1605	*A. ferreus*	S Pistol River Road and H101, Curry Co., OR	12‐18‐1995
1626–1634	*A. ferreus*	S Pistol River Road and H101, Curry Co., OR	1‐26‐1996
1664, 1666, 1668–1681	*A. ferreus*	S of H101 Jtn on Carpenterville Rd, Curry Co., OR	3‐2‐1996
1749–1758	*A. ferreus*	S of H101 Jtn on Carpenterville Rd, Curry Co., OR	8‐3‐1996
1772–1776	*A. ferreus*	S of H101 Jtn on Carpenterville Rd, Curry Co., OR	10/26,27/1996
1469–1480, 1482–83	*A. flavipunctatus*	Along H128, W of church, Philo, Mendocino Co., CA	3‐8‐1994
1481	*A. lugubris*	Along H128, W of church, Philo, Mendocino Co., CA	3‐8‐1994
1484–1489	*A. lugubris*	Neighborhood of 600‐700s Calmar, Oakland, Alameda Co., CA	3‐8‐1994
1490 Oakland Rose Garden	*A. lugubris*	Near Oakland Rose Garden, Oakland, Alameda Co., CA	3‐8‐1994
1546–1549, 1551–1554	*A. lugubris*	Neighborhood of 600‐700s Calmar, Oakland, Alameda Co., CA	2‐11‐1995
1606, 1607, 1610	*A. lugubris*	north Berkeley neighborhoods, Alameda Co., CA	1/20,21/1996
1608–09, 1611–14	*A. lugubris*	North Berkeley neighborhoods, Alameda Co., CA	1‐21‐1996
1687, 1691	*A. lugubris*	Neighborhoods of 600s and 700s Calmar and 600s Rosal, Oakland, Alameda Co, CA	3‐23‐1996
1788–1793	*A. lugubris*	Neighborhood of 600‐700s Calmar, Oakland, Alameda Co., CA	11‐2‐1996
1795–1802	*A. lugubris*	Neighborhood of 600‐700s Calmar, Oakland, Alameda Co., CA	2‐8‐1997
1339–1347, 1349–1360	*A. hardii*	FS Rd 64, 2 mi from Highway 6563, T17S, R.12E center of section 36, Otero Co. NM	8‐9‐1992
1522–1540	*A. hardii*	FS road 64 off of NM road 6563, S of Cloudcroft, T17S R12E center of section 36, Otero Co, NM	6‐10‐1994
1708–1710, 1712, 1714–1720	*A. hardii*	FS road 64 off of NM road 6563, S of Cloudcroft, T17S R12E center of section 36, Otero Co, NM	7‐3‐1996
1730–1736	*A. hardii*	On 64 after turnoff of NM road 6563, S of Cloudcroft, Otero Co, NM	7‐4‐1996
1815–1828, 1830–1834	*A. hardii*	On 64 after turnoff of NM road 6563, S of Cloudcroft, Otero Co, NM	5‐23‐1997
1836–1847	*A. hardii*	On 64 after turnoff of NM road 6563, S of Cloudcroft, Otero Co, NM	26‐07‐1997
1568	*P. elongatus*	Along Hunter Creek, on FS road 3680. T37S, R13W, E center of section 8. Curry Co., OR	18‐11‐1995
1620–25	*P. elongatus*	Near Lobster Creek Bridge, N side Rogue River, Curry Co., OR	25‐01‐1996
1661–1663	*P. elongatus*	E of Hwy 101 on Chetco River Rd, North bank, Brookings, Curry Co., OR	02‐03‐1996
1777–82	*P. elongatus*	Winchuk campground, S of Brookings, Curry Co., OR	10/26,27/1996
1783–1787	*P. elongatus*	Lobster Creek Bridge area, N bank of Rogue River, Curry Co., OR	10/26,27/1996

^a^ ID numbers are NLS numbers; will be accessioned into the Museum of Vertebrate Zoology, UC Berkeley in 2025.

^b^ Date of collection and blood sampling.
